# Crosstalk within a brain-breast-bone axis regulates mineral and skeletal metabolism during lactation

**DOI:** 10.3389/fphys.2023.1121579

**Published:** 2023-02-16

**Authors:** Diana Athonvarangkul, John J. Wysolmerski

**Affiliations:** Section of Endocrinology and Metabolism, Department of Internal Medicine, Yale University School of Medicine, New Haven, CT, United States

**Keywords:** lactation, bone loss, calcium, mammary gland, osteocytic osteolysis, calcium sensing receptor, PTHrP, estrogen deficiency

## Abstract

To support the increased calcium demands for milk production during lactation, a dramatic and reversible physiological response occurs to alter bone and mineral metabolism. This coordinated process involves a brain-breast-bone axis that integrates hormonal signals that allow for adequate calcium delivery to milk yet also protects the maternal skeletal from excessive bone loss or decreases in bone quality or function. Here, we review the current knowledge on the crosstalk between the hypothalamus, mammary gland, and skeleton during lactation. We discuss the rare entity of pregnancy and lactation associated osteoporosis and consider how the physiology of bone turnover in lactation may impact the pathophysiology of postmenopausal osteoporosis. Further understanding of the regulators of bone loss during lactation, particularly in humans, may provide insights into new therapies for osteoporosis and other diseases of excess bone loss.

## Introduction

The synthesis and secretion of milk is an essential aspect of mammalian reproduction as milk supplies the complete nutritional requirements for neonatal survival and growth. Given that the neonatal period represents the most rapid period of skeletal growth, milk must supply large amounts of calcium. Exporting calcium to offspring in this manner stresses maternal calcium and bone metabolism, requiring adaptations to avoid hypocalcemia. Meeting this challenge requires activation of a brain-breast-bone circuit in lactating mothers that coordinates changes in systemic hormones, the availability of dietary calcium, skeletal turnover and calcium transport into milk (Wysolmerski, 2010). Classically, it has been taught that intestinal calcium absorption satisfies extra calcium needs during pregnancy and that increased bone resorption is the main source of calcium for milk production during lactation (Kovacs, 2016). In truth, both mechanisms provide calcium for the fetus and neonate during reproductive cycles and likely provide redundancy to ensure adequate supplies of calcium for the synthesis of new bone needed for linear growth of the offspring both in the latter part of gestation as well as during preweaning growth. Therefore, lactation is associated with rapid bone loss that, fortunately, is fully restored after weaning. Milk production does not usually result in permanent bone loss and does not predispose to fractures either during reproductive life or to post-menopausal osteoporosis in later life. However, in very rare instances, women can develop pregnancy and lactation-associated osteoporosis (PLO) accompanied by fractures during the third trimester of pregnancy or during breastfeeding (Smith et al., 1995; Stumpf et al., 2007; Pola et al., 2016; Hadji et al., 2017; Laroche et al., 2017; Kyvernitakis et al., 2018; Hardcastle et al., 2019; Tuna et al., 2020).

Over the past few decades, investigators have described many aspects of the integrated physiology of bone and mineral metabolism associated with reproductive cycles, but there remain many unanswered questions. Clinical studies in nursing women have measured bone density and bone turnover markers, but few mechanistic studies (e.g., involving bone biopsies) exist. As a result, our current models rely mostly on data derived from rodent models. However, there are key differences in the physiological response to support a single human baby compared to a litter of 8–12 rodent offspring. With that caveat in mind, in this review, we will discuss elements of the brain-breast-bone circuit that regulates bone and mineral metabolism during lactation and post-weaning in rodent models, review the changes described in women, and highlight unanswered questions for future research. Finally, we will underscore how the pathophysiology of post-menopausal osteoporosis represents the reactivation of physiologic responses to the demands of milk production.

### Bone loss during lactation

Bone loss during lactation has been extensively documented in experimental mice and rats. Mice lose between 20% and 33% of their bone mass as assessed by dual energy X-ray absorptiometry (DXA) at different skeletal sites as compared to age-matched nulliparous controls (Kovacs and Kronenberg, 1997; Woodrow et al., 2006; Qing et al., 2012). Loss of bone mineral density (BMD) by DXA has been noted to be maximal at mid-lactation (12 days *postpartum*), but then plateaus between 12 and 18 days of lactation. In mice, greater losses in BMD during lactation occur at trabecular-rich sites (spine −10.9%, distal femur −12.6%, and proximal tibia −19.9%) compared to less significant losses in BMD at predominantly cortical sites (distal tibia and middle tibia, −5% each) (Zeni et al., 1999). Changes in BMD are paralleled by similar reductions in trabecular volume, trabecular number and trabecular thickness when bones are examined histologically (VanHouten and Wysolmerski, 2003). In addition, micro-computed tomography (CT) imaging of bone has demonstrated deterioration in trabecular microarchitecture as well as a decrease in cortical thickness and an increase in cortical porosity in lactating as compared to nulliparous mice (Liu et al., 2012). Similarly, studies in rats have demonstrated 15%–35% reductions in BMD at the spine, hip and total body as well as reductions in trabecular thickness, trabecular number and cortical thickness in lactating versus nulliparous animals (Warnock and Duckworth, 1944; Ellinger et al., 1952; Miller et al., 1986; Tojo et al., 1998; Honda et al., 2000). The degree of bone loss is modulated by suckling intensity as the duration of lactation and differences in the number of suckling pups have been shown to affect the degree of bone loss (Peng et al., 1988). The dramatic changes in bone mass and structure are accompanied by changes in the material properties and mechanical strength of bone. Studies on mouse femurs or vertebrae have documented reductions in the elastic modulus (de Bakker et al., 2017; Kaya et al., 2017), while 3-point bending studies of femurs and compression studies on vertebrae have demonstrated reductions in stiffness and ultimate load at mid-lactation in mice and rats (Vajda et al., 2001; Kaya et al., 2017). Thus, bone loss during lactation is associated with clear changes in bone strength and mechanical susceptibility to fracture that are reversible post weaning (see below).

Bone loss is also well documented in women, although the rates and magnitude of skeletal catabolism are lower in nursing humans than in lactating rodents. Women typically lose between 5% and 10% of bone mineral content (BMC) with 3–6 months of exclusive breastfeeding, which is fully recovered within 6–12 months after weaning (Kalkwarf and Specker, 1995; Polatti et al., 1999; Kovacs, 2011; Brembeck et al., 2015; Bjørnerem et al., 2017). As in rodents, women lose more bone at trabecular-rich sites such as the spine and hip (Laskey et al., 1998), with a mean loss of 4% in lumbar spine bone mineral density after 3 months of breastfeeding (Kalkwarf and Specker, 1995; Laskey et al., 1998; Polatti et al., 1999). The magnitude of bone loss is positively correlated with the amount of milk production, with women nursing twins or triplets losing more bone than women nursing one baby (Kalkwarf and Specker, 1995; Brembeck et al., 2015; Bjørnerem et al., 2017), and greater bone loss occurring in women with exclusive versus intermittent breastfeeding (Bjørnerem et al., 2017) or a longer duration of breastfeeding (More et al., 2001; Brembeck et al., 2015). In contrast, women who feed their child with formula do not experience a significant decline in BMD, demonstrating that *postpartum* bone loss is a consequence of lactation rather than of delivery itself (Kalkwarf and Specker, 1995; Laskey et al., 1998; Polatti et al., 1999). Studies of bone microstructure have been performed in humans using high-resolution peripheral quantitative computed tomography (HR-pQCT) and document increased cortical porosity and declines in cortical thickness, trabecular number, bone volume, bone mineral density, and mineralization (Brembeck et al., 2015; Bjørnerem et al., 2017). Bone loss during lactation is obligate and cannot be prevented or significantly reduced by increasing dietary calcium in nursing humans. Both randomized and observational studies have shown that neither high nor low dietary calcium intake influence loss of BMD in nursing women, although low calcium diets have been shown to dramatically accelerate bone loss in lactating rodents (Ellinger et al., 1952; Bawden and McIver, 1964; Rasmussen, 1977a; Rasmussen, 1977b; Cross et al., 1995; Kalkwarf et al., 1997). Finally, there are no studies of bone mass by histomorphometry in nursing women nor are there any direct measurements of bone strength in women. Bone strength is likely to be reduced even though women do not typically fracture while nursing except in very rare cases of pregnancy and lactation associated osteoporosis (Smith et al., 1995; Stumpf et al., 2007; Pola et al., 2016; Hadji et al., 2017; Laroche et al., 2017; Kyvernitakis et al., 2018; Hardcastle et al., 2019; Tuna et al., 2020), which will be discussed in a subsequent section.

### Skeletal remodeling during lactation

Rapid bone loss is associated with increased bone turnover in lactating rats and mice. Studies in rodents using biochemical markers of bone resorption and bone formation have shown that rates of both bone formation and bone resorption are elevated (VanHouten and Wysolmerski, 2003; Woodrow et al., 2006; Ardeshirpour et al., 2007; Kirby et al., 2011; Bornstein et al., 2014; Gillies et al., 2018). In addition, both static and dynamic histomorphometry has demonstrated increased numbers of osteoclasts, increased numbers of osteoblasts and increased rates of bone formation in lactating animals despite the reductions in bone mass itself. Bone resorption occurs primarily along trabecular surfaces and endocortical surfaces of long bones. Bone formation is observed in the same areas, although bone resorption clearly outpaces bone formation during this period of rapid bone loss. The increases in osteoclast and osteoblast numbers on bone surfaces are also mirrored by increased numbers of osteoclast and osteoblast precursors that can be recovered from the bone marrow of lactating versus nulliparous mice (Kirby et al., 2011).

As with bone density data, bone turnover studies in nursing women largely mirror the findings in experimental animals. Circulating levels of bone formation markers, such as procollagen 1 Intact N-terminal propeptide (P1NP) or osteocalcin, as well as circulating levels of bone resorption markers, such as collagen type 1 c-telopeptide (CTX) and n-telopeptide (NTX), have been shown to be elevated in nursing women (Sowers et al., 1995). There are no studies that have performed bone biopsies in normal lactating women, so we do not have histomorphometric studies to correlate with bone turnover markers. However, in other settings, osteoclast and osteoblast numbers as well as bone formation rates generally parallel bone turnover markers in women (Parfitt et al., 1987; Eriksen et al., 1993). Furthermore, HR-pQCT data showing trabecular thinning and loss along with increased cortical porosity support increased rates of bone resorption both on surfaces and within the cortices of long bones in lactating women (Brembeck et al., 2015; Bjørnerem et al., 2017).

In addition to increased rates of osteoclastic resorption on bone surfaces, lactation is also associated with an increase in osteocyte lacunar-canalicular remodeling, also referred to as osteocytic osteolysis. The ability of osteocytes to resorb minerals from the surrounding bone was first described by Rigal and Vignal who observed widened lacunae around osteocytes in 1881 (Rigal and Vignal, 1881). Subsequently, von Recklinghausen hypothesized that osteocytes could digest their perilacunar matrix during times of increased mineral demand (Von Recklinghausen, 1910). Although the concept never completely disappeared, in the ensuing decades, the validity of osteocytic osteolysis was questioned as an artifact from sample processing and bone resorption was thought to be solely due to the activity of surface osteoclasts (Parfitt, 1977; van der Plas et al., 1994). However, a series of reports over the last decade have again highlighted the potential importance of osteocytic osteolysis during lactation. Qing and colleagues convincingly demonstrated that, during lactation, osteocytes express osteoclast-like genes and enzymes that allow them to demineralize and resorb the bone surrounding their lacunae as well as around the canalicular network (Qing et al., 2012). As a result, the osteocyte lacunae and canaliculi increase in size at mid-lactation as compared with nulliparous controls. Subsequent studies have shown that osteocytes upregulate proton pumps that allow them to acidify their microenvironment (Jähn et al., 2017) and they also secrete Cathepsin K and matrix metallopeptidase 13 (MMP13), which are required for enlargement of the lacunae (Tang et al., 2012; Lotinun et al., 2019). Osteocyte-specific deletion of the Type 1 PTH/PTHrP receptor (PTHR1) reduces the amount of bone mineral density lost during lactation by 50%. Although DMP1-cre used to target osteocytes in this study may also affect endosteal osteoblasts, results from the deletion of PTHR1 in osteocytes suggest that osteocytic osteolysis, directly or indirectly, may contribute to net bone loss during lactation (Qing et al., 2012). Interestingly, deletion of the PTHR1 or cathepsin K in osteocytes not only blunts the changes in lacunar size during lactation but also reduced osteoblast and osteoclast numbers and activity associated with lactation (Qing et al., 2012; Lotinun et al., 2019). Osteocytes are well known to respond to mechanical forces and to regulate the activity of surface bone cells influencing overall bone turnover. Therefore, it is likely that the phenotypic change in osteocyte differentiation during lactation as well as the alterations in lacunar and canalicular volumes associated with osteocytic osteolysis, modulate how these cells integrate hormonal changes, local cytokine alterations, and the effects of microstrain related to mechanical forces. In a recent review, Liu and colleagues suggest that the increased osteocytic fluid space resulting from osteocytic osteolysis might make osteocytes more sensitive to mechanical signals, an idea that is partly supported by animal studies demonstrating that increased loading can reduce bone loss during lactation and enhance bone recovery after weaning (Liu et al., 2019). This is an interesting area that underscores the interconnectedness of bone cell activity and structural changes in the skeleton. A limitation of studying rodent bone is the lack of osteons and the Haversian canal system. Unfortunately, to date, there are no human studies on osteocytic osteolysis in lactation, so it is unclear whether similar changes in the osteocyte lacunar-canalicular network occur in nursing women.

### Skeletal recovering in the post-weaning period

Fortunately, bone loss during pregnancy and lactation is followed by a remarkable period of bone recovery after weaning that appears to restore bone mass and strength back to its pre-pregnancy baseline (Kovacs and Kronenberg, 1997; Bowman et al., 2002; Miller et al., 2005; Ardeshirpour et al., 2007). Rats and mice recover the bone that was lost during lactation within 4 weeks after either forced withdrawal of pups or “natural weaning” (Ardeshirpour et al., 2007; Liu et al., 2012). As detailed previously, both osteoblast and osteoclast cell numbers and activity are increased during lactation. Mechanistically, weaning triggers a sudden and coordinated wave of osteoclast apoptosis, leading to a dramatic reduction in osteoclastic bone resorption (Ardeshirpour et al., 2007; Miller and Bowman, 2007). At the same time, bone formation rates are either unaffected or increase, resulting in a period of unopposed bone formation. Data differ on whether osteoblast numbers increase further or remain at their already increased levels upon weaning. Ardeshirpour and colleagues found that, in mice, osteoblast numbers and bone formation rates at 3 days following forced weaning were no different than at mid-lactation. However, the numbers of osteoblast progenitors within the bone marrow at 3 days were further increased over the already increased numbers during lactation (Ardeshirpour et al., 2007). In rats, Bowman and Miller showed that an increase in the proliferation of osteoblast progenitors after weaning gives rise to an expansion of the osteoblast population (Miller and Bowman, 2007). Within 25 h after weaning, they found a 20%–24% increase in newly formed osteoblasts and that 75% of bone surfaces became covered with osteoid. From day 2 to day 7 post-weaning, there was a doubling of bone formation indices by histomorphometry (Miller et al., 2005). This anabolic phase of bone recovery may rely on prior bone resorption and/or bone loss. Our laboratory reported that administration of osteoprotegerin (OPG), a decoy RANKL receptor that inhibits bone resorption, during lactation not only lowered osteoblast numbers during lactation but also prevented the typical increase in bone formation post-weaning, (Ardeshirpour et al., 2015). In contrast, Wendelbow and colleagues found that inhibiting bone resorption with zoledronic acid, a bisphosphonate that is a pharmacological inhibitor of bone resorption, prevented bone loss during lactation but, in contrast to our findings, bone mass increased over baseline after weaning (Wendelboe et al., 2016). Comparing these two studies suggests that RANKL-RANK signaling may somehow be involved both in regulating bone resorption during lactation and in allowing its recovery after weaning. Finally, weaning also leads to a reversal in the changes that occur in the osteocyte lacunar-canalicular network during lactation. Upon weaning, osteocytes rapidly lose their osteoclast-like properties (Qing et al., 2012) and revert to an osteoblast-like phenotype with the ability to re-mineralize their lacunae as evidenced by dual tetracycline labeling (Qing and Bonewald, 2009). The enlarged osteocyte lacunae that occur during lactation completely revert to their baseline volumes within 1-week post-weaning (Kaya et al., 2017).

Women also gain bone rapidly after cessation of lactation. Most studies have found that, after they stop nursing, women experience a complete recovery of BMD to baseline over a period of 6 months (Kalkwarf and Specker, 1995; Laskey et al., 1998; Polatti et al., 1999; Cooke-Hubley et al., 2017). Mirroring the more rapid rate of bone loss at trabecular-rich sites, it has been suggested that recovery at the trabecular rich spine occurs faster than at cortical sites. Not all studies show a complete recovery of bone mass after weaning in women. One study of 10 adolescent mothers who habitually consumed low calcium diets reported incomplete recovery with a lower accretion rate than predicted for age-matched controls (Bezerra et al., 2004). In addition, studies using HR-pQCT, have suggested that microarchitecture may not completely revert to the nulliparous state at all sites (Brembeck et al., 2015). In a longitudinal study with a median follow up period of 3.6 years, cortical porosity remained higher while mineralization density and trabecular number remained lower in women who had lactated compared to controls who were non-pregnant non-lactating premenopausal women (Bjørnerem et al., 2017).

There is no evidence that unresolved microarchitectural changes from multiple cycles of pregnancy and lactation lead to cumulative damage that might increase the risk for osteoporosis and fragility fracture. In fact, epidemiological studies suggest that bone loss during lactation does not predispose to an increased risk of osteoporosis. There is a neutral or protective effect of lactation on peak bone mass, bone density and fracture risk, including in a large study of twins with discordant lactation history (Paton et al., 2003; Chantry et al., 2004; Specker and Binkley, 2005; Kovacs, 2016; Song et al., 2017). In the NHANES III study, women aged 20–25 years who breastfed as adolescents had higher BMD than their contemporaries who did not breastfeed or were nulliparous (Chantry et al., 2004). Studies looking at long term clinical outcomes mostly show that parity and lactation are not associated with any increase in fracture risk (Specker and Binkley, 2005; Watts et al., 2021). However, there may be cultural or genetic differences that modulate whether breastfeeding increases the risk for later osteoporosis. For example, in a study of a subgroup of 1,342 women from South Korea who were part of the 2010 Korea National Health and Nutrition Examination Survey, Yeo and colleagues found that a history of breastfeeding, especially for over 1 year, was an independent predictor of lower spine bone mineral density after menopause (Yeo et al., 2016). More studies need to be performed to know whether this study is an outlier or whether there is a true difference in East Asian women as compared to other racial groups. In addition, there are rare women who develop pregnancy and lactation associated osteoporosis, who may have predisposing factors or clinical circumstances that result in breastfeeding compromising bone strength and quality after parturition and beyond.

Exactly what stimulates skeletal recovery post-weaning remains unclear. Animal studies have shown that the classic calciotropic hormones such as parathyroid hormone (PTH), PTH-related peptide (PTHrP), calcitonin, vitamin D, vitamin D receptor and estrogen are not required for the recovery of ash weight, BMC or bone strength following weaning (Kovacs, 2016). Adequate calcium intake is necessary as a restricted calcium diet prevented bone recovery until after the diet was normalized (Hagaman et al., 1990). A recent study showed that osteoblasts can lay down osteoid independent of vitamin D but adequate calcium in the diet is required for the immature bone to become mineralized during the post-weaning period (Ryan et al., 2022). A low protein diet during lactation and the post-weaning periods appears to enhance lactation-associated bone loss and impede post-weaning bone recovery. Osteoblasts from animals on a low protein diet showed reduced mineralization capacity *in vitro* (Kanakis et al., 2020)*.* Not surprisingly, a comparison of the tibial transcriptomes between mice after weaning versus pre-pregnancy, revealed hundreds of differentially regulated genes involved in osteoblastogenesis, osteoclast inhibition, and the regulation of energy metabolism (Collins et al., 2013). Clearly, much more work is required to understand the molecular triggers that lead to osteoclast apoptosis and the stimulation of osteoblast differentiation and activity.

Data on the factors that may trigger post-weaning skeletal recovery in women are even more scarce. While resumption of menses with rising estradiol levels and falling PTHrP levels have been suggested to slow osteoclastic bone resorption at the weaning transition, there is no evidence that these changes stimulate bone formation. What is clear, and what corroborates findings from animal models, is that PTH is not required for skeletal recovery following lactation as women with hypoparathyroidism can recover bone mass in the lumbar spine and femoral neck normally after weaning (Segal et al., 2011).

### Hormonal regulation of bone turnover during lactation

**FIGURE 1:**
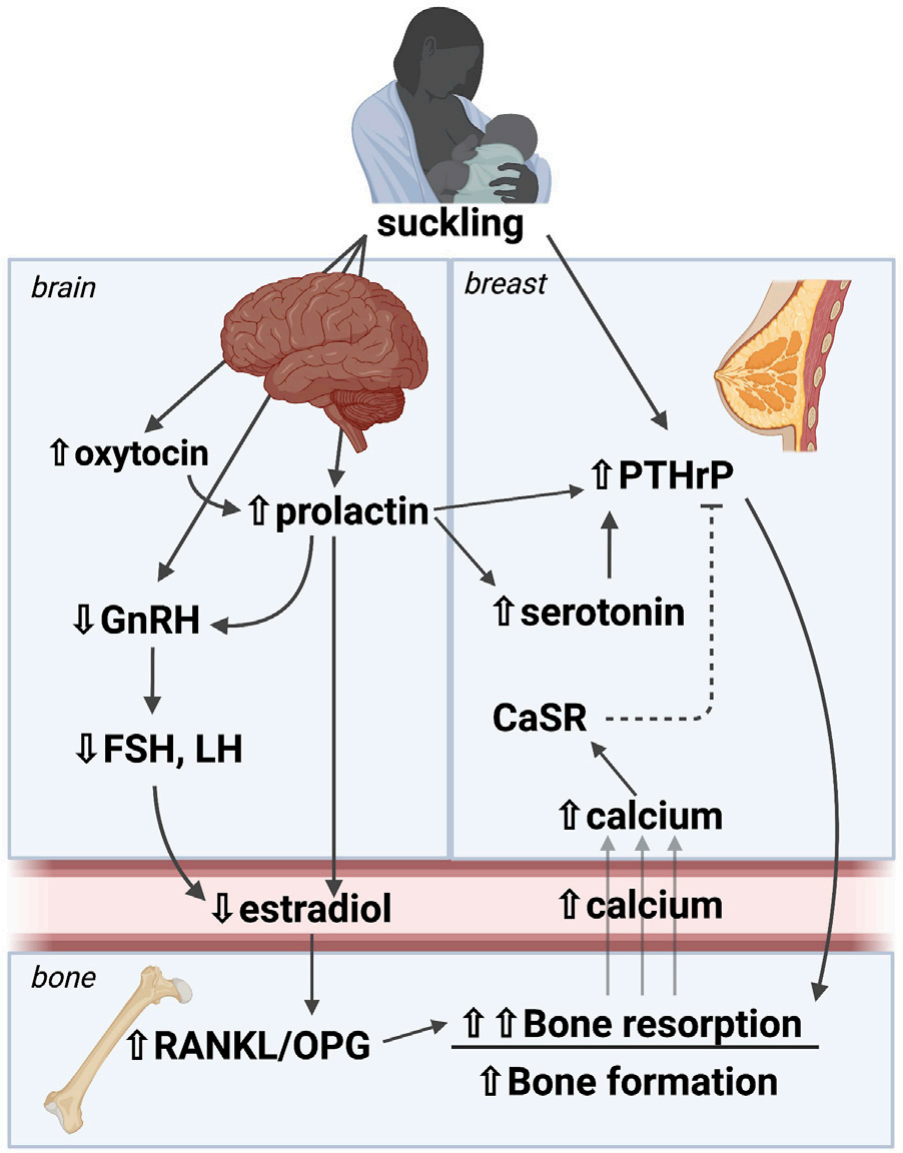
A brain-breast-bone axis regulates skeletal and mineral metabolism during lactation. Suckling stimulates afferent nerves from the mammary gland, which relay through the brainstem to the hypothalamus to inhibit GnRH production, and to stimulate prolactin and oxytocin secretion. The resulting hypogonadotropic hypogonadism leads to estrogen deficiency. The mammary gland secretes PTHrP into the circulation in response to suckling and increased circulating prolactin, effects that can be inhibited by stimulation of the calcium-sensing receptor (CaSR) by calcium delivery to the mammary gland. The combined effect of low estradiol and elevated PTHrP levels in lactation promote osteoclastic bone resorption as well as osteocytic osteolysis. These changes are the best studied to date, but they alone do not fully recapitulate the bone loss that occurs naturally with lactation, indicating that there are likely additional local or systemic factors that are involved in this process. PTHrP: Parathyroid hormone-related peptide, GnRH: Gonadotropic releasing hormone, FSH: Follicle stimulating hormone, LH: Leutenizing hormone, CaSR: Calcium sensing receptor, RANKL: Receptor activator of NF-KB ligand, OPG: osteoprotegerin. Image made with BioRender.

The regulation and coordination of calcium and bone metabolism during lactation relies on interactions among the breast, brain and bone that regulate systemic bone turnover (Figure 1). Individual hormonal regulators will be discussed in depth below, but, in overview, suckling stimulates the pituitary to release prolactin and oxytocin. In addition, suckling stimulates afferent nerves from the mammary gland, which relay through the brainstem to the hypothalamus and inhibit gonadotropic releasing hormone (GnRH) production. This, in turn, suppresses estradiol production leading to hypogonadotropic hypogonadism, which is reinforced by elevations in circulating prolactin. The mammary gland also secretes PTHrP into the circulation in response to suckling and increased circulating prolactin, effects that can be inhibited by stimulation of the calcium-sensing receptor (CaSR) by calcium delivery to the mammary gland. Clinical and animal studies confirm that the combined effect of low estradiol and elevated PTHrP levels during lactation promotes osteoclastic bone resorption as well as osteocytic osteolysis (Zinaman et al., 1990; Sowers et al., 1996; Kovacs and Kronenberg, 1997; Kalkwarf, 1999; Kovacs, 2001; VanHouten et al., 2003; VanHouten and Wysolmerski, 2003; Ardeshirpour et al., 2010). Furthermore, both prolactin and oxytocin have been shown to have effects on bone. However, these changes alone do not fully recapitulate the bone loss that occurs naturally with lactation, indicating that there are likely additional local or systemic factors that are involved in this process, but have yet to be defined (Ardeshirpour et al., 2010). There are a paucity of data on the effect of this breast-brain-bone circuit in post-weaning skeletal recovery, so we will focus our discussion on bone loss during lactation.

### Estrogen deficiency

Estrogen is a key regulator of bone remodeling, and osteoclasts, osteocytes and osteoblasts all express the estrogen receptor (Riggs, 2000). The bone sparing effects of estrogen on osteoclasts and osteocytes is primarily mediated by estrogen receptor alpha (Movérare et al., 2003; Sims et al., 2003; Vandenput and Ohlsson, 2009; Windahl et al., 2013). The effects of estrogen receptor beta in bone cells may be to antagonize the effects of estrogen receptor alpha, but its role is less well characterized (Windahl et al., 1999; Windahl et al., 2001; Sims et al., 2002; Lindberg et al., 2003). One of the most important actions of estrogen on bone involves the osteoprotegerin (OPG)/receptor activator of NF-KB ligand (RANKL) system. RANKL is a cytokine expressed by osteoblasts, osteocytes and bone marrow cells, that is essential for osteoclast differentiation, activation, and survival (Kong et al., 1999). OPG, which is also secreted by osteoblasts, osteocytes and marrow stromal cells, is a soluble decoy receptor that binds RANKL to inhibit osteoclastogenesis, protecting the skeleton from excess bone resorption (Simonet et al., 1997; Lacey et al., 1998). RANKL acts through its receptor RANK (Receptor Activator of NF-kB) which is expressed on osteoclast precursors and mature osteoclasts (Li et al., 2000). RANKL, RANK and OPG are non-redundant and essential osteoclast factors. The ratio of RANKL/OPG determines the recruitment and differentiation of precursors to become osteoclasts, the resorbing activity of mature osteoclasts, and the lifespan of resorbing osteoclasts. In studies of ovariectomized female mice, estrogen withdrawal increases RANKL expression and decreases OPG expression leading to an increase in the RANKL/OPG ratio and an increase in the numbers and activity of osteoclast and, subsequently, increased bone resorption (Streicher et al., 2017). The importance of RANKL in the pathogenesis of estrogen-deficiency induced bone loss has been translated into therapeutic use of a humanized monoclonal antibody against RANKL, denosumab, for the treatment of postmenopausal osteoporosis (Zhang et al., 2020).

Lactation represents the only period of prolonged estrogen deficiency naturally occurring during the reproductive lifespan. Low estrogen results from hypogonadotropic hypogonadism that occurs in response to suckling. Afferent nerves from the breast relay the suckling reflex through the hindbrain and to the hypothalamus, where it leads to an increase in the activity of neuropeptide Y (NPY)/agouti-related protein (AGRP)-positive, melanin concentrating hormone (MCH)-positive and oxytocin-positive neurons as well as a decrease in kisspeptin 1 (Kiss1) secretion (Brown et al., 2014). The increase in NPY is also reinforced by the reductions in circulating insulin and leptin that occur during lactation (Brogan et al., 1999; Xu et al., 2009). The changes in these neurotransmitters lead to a suppression of pulsatile GnRH secretion, low LH and FSH levels, and decreased ovarian estrogen secretion. Finally, the increase in prolactin secretion in response to suckling reinforces the suppression of GnRH secretion by inhibiting the production and secretion of Kiss1. In women, the degree of GnRH suppression and hypoestrogenemia is dependent on the intensity of the suckling signal as well as its timing–as nursing during nightime is more effective at suppressing GnRH than it is during daytime (Smith and Grove, 2002; Smith et al., 2010; Brown et al., 2014).

In lactating mice, estrogen levels are inversely associated with rates of bone resorption and estrogen replacement reduces bone loss (VanHouten and Wysolmerski, 2003). In human studies, bone loss correlates with the duration of postpartum amenorrhea, which reflects an ongoing low estrogen state (Sowers et al., 1993; Caird et al., 1994; Kalkwarf and Specker, 1995; Honda et al., 1998; Kolthoff et al., 1998; Polatti et al., 1999). However, clinical and animal data indicate that estrogen deficiency is not the only cause of lactational bone loss. Bone loss also varies with suckling intensity. For instance, mice suckling larger litters lose more bone and, likewise, so do mothers nursing twins as compared to mothers nursing single infants (Garner et al., 1987; Peng et al., 1988; Laskey et al., 1998; Kovacs, 2016). Whether this is related to more effective suppression of estrogen levels is not known. In addition, women treated with GnRH analogues for endometriosis, fibroids and severe acne over 6 months have lower circulating levels of estrogen than lactating women, but experience less bone loss (Rico et al., 1993; Fogelman et al., 1994; Orwoll et al., 1994; Uemura et al., 1994; Howell et al., 1995; Newhall-Perry et al., 1995; Roux et al., 1995; Paoletti et al., 1996; Taga and Minaguchi, 1996). Rodent studies corroborate these findings and have shown that lactating animals have greater bone loss than animals rendered estrogen deficient by ovariectomy (Alles et al., 2010; Ardeshirpour et al., 2010; Duque et al., 2011). Estrogen replacement in lactating mice reduced bone loss by ∼60% but did not completely prevent it which is consistent with the clinical observation that nursing women can continue to lose bone after return of menses (Holmberg-Marttila et al., 2003; VanHouten and Wysolmerski, 2003). Together, these studies show that low estrogen works in tandem with other lactation hormones or factors to promote bone loss.

### Parathyroid-related peptide hormone (PTHrP): A signal from the mammary gland to bone

Parathyroid hormone-related protein (PTHrP) shares sequence homology with parathyroid hormone (PTH) at its amino terminus and signals through the common Type 1 PTH/PTHrP receptor (PTHR1). It was discovered as a cause of humoral hypercalcemia of malignancy (HHM), a common complication of many cancers including breast cancer (Burtis et al., 1987; Moseley et al., 1987; Strewler et al., 1987). Subsequent studies demonstrated that PTHrP is secreted by normal breast epithelial cells during lactation, where it becomes a key player regulating systemic calcium and bone metabolism (VanHouten et al., 2003). Circulating levels of PTHrP are very low or unmeasurable in nulliparous mice. However, they become elevated during lactation and are further increased by suckling and prolactin in both mice and in women (Rakopoulos et al., 1992; Lippuner et al., 1996; Sowers et al., 1996; VanHouten et al., 2003; VanHouten, 2005; Ardeshirpour et al., 2010; Vanhouten and Wysolmerski, 2013). Circulating PTHrP levels have been shown to correlate positively with bone resorption markers and negatively with bone mass in mice at mid-lactation (VanHouten and Wysolmerski, 2003). PTHrP levels in nursing women have also been shown to correlate with the degree of bone loss and higher bone turnover during lactation (Lippuner et al., 1996; Sowers et al., 1996). These studies suggest that increased circulating levels of PTHrP might synergize with estrogen deficiency to promote bone loss during lactation.

In order to test whether breast-derived PTHrP acts as a hormone to increase bone resorption and cause bone loss during lactation, VanHouten and colleagues disrupted the mouse *Pthlh* (PTHrP) gene specifically in mammary epithelial cells during lactation. Deletion of PTHrP from mammary glands eliminated PTHrP from the circulation as well as from milk, demonstrating that the mammary gland is the source of circulating PTHrP during lactation. Bone resorption assessed by biochemical markers and histomorphometry was reduced by nearly 50% while bone density measured by DXA was significantly higher in the absence of mammary gland PTHrP production as compared to controls (VanHouten et al., 2003).

A study by Ardeshirpour and colleagues found that infusion of PTHrP into nulliparous mice increases bone resorption rates and amplifies the bone loss associated with ovariectomy or with treatment with a GnRH analogue to mimic the hypogonadotropic hypogonadism associated with lactation. Nulliparous mice were administered PTHrP *via* a continuous mini-osmotic pump, either alone, or with treatment of the GnRH agonist leuprolide or surgical ovariectomy to lower estrogen levels. Circulating estrogen levels in mice that were treated with leuprolide, that underwent ovariectomy, and that lactated were lower than in randomly cycling virgin mice and comparable among the hypogonadotropic states. Compared to lactating animals that lost 23% of BMD in the spine and 16% in the femur over 10 days of lactation, PTHrP decreased BMD by 4%–5%, whereas leuprolide had a neutral to slightly positive effect on BMD. The combination of PTHrP *via* infusion and low estrogen levels did amplify the bone loss, but the overall magnitude of bone loss did not equal that of natural lactation. Analysis of histomorphometry found that leuprolide alone increased osteoclast numbers, whereas PTHrP alone or in combination with leuprolide increased both osteoclast and osteoblast numbers (Ardeshirpour et al., 2010). A clinical study by Horowitz et al. also showed that PTHrP infusions could cause an increase in bone turnover and suppression of bone formation, as assessed by NTX, CTX, P1NP and bone-specific alkaline phosphatase in the serum (Horwitz et al., 2011). Clearly, infusion studies in mice or in humans do not recapitulate true lactation physiology (Ardeshirpour et al., 2010; Horwitz et al., 2011). For instance, in the absence of lactation the calcium released by the skeleton could not go into milk and hypercalcemia ensued (Horwitz et al., 2011) in both humans and mice. Nevertheless, these studies show that estrogen deficiency and PTHrP can work in conjunction to activate bone loss in lactation, but they also suggest that there may be significant contributions from other local or systemic factors to the full effects of lactation on the skeleton.

### Calcium sensing receptor

**FIGURE 2:**
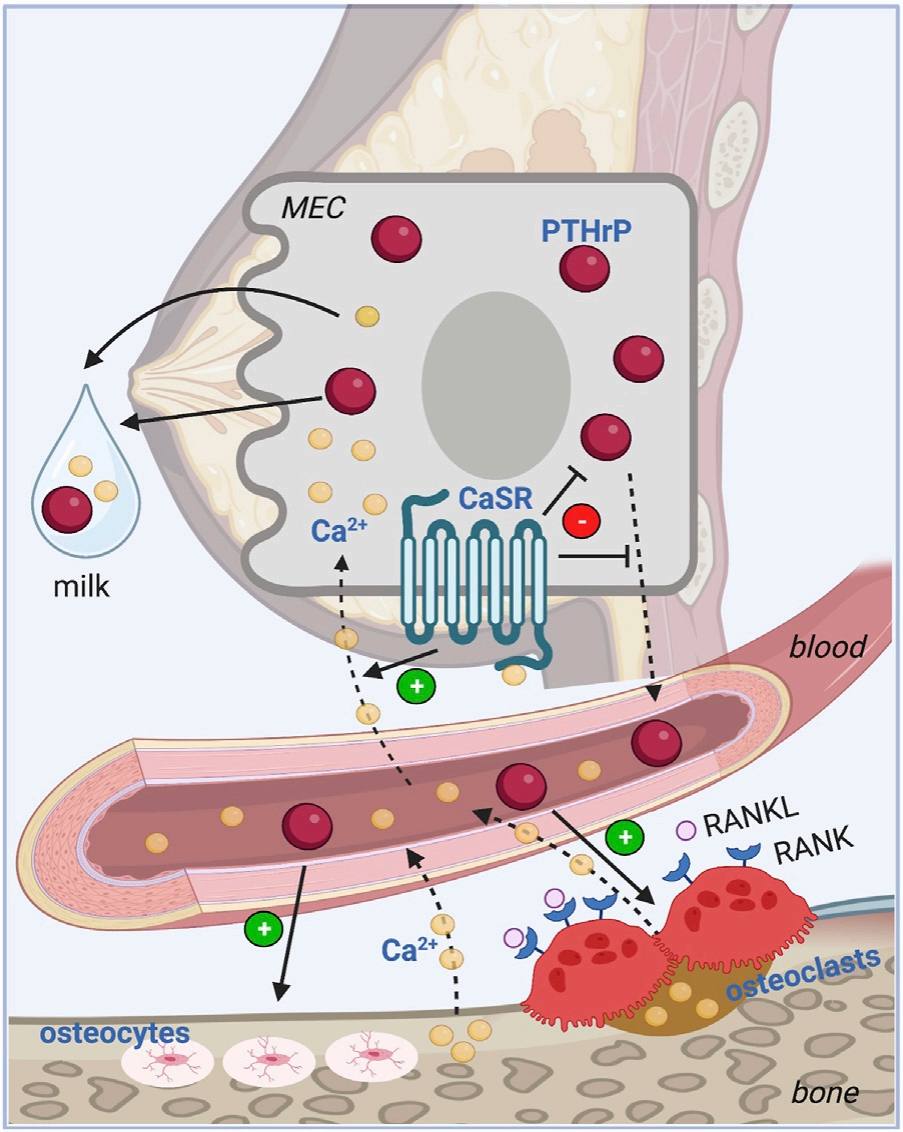
Calcium sensing feedback loop between bone and breast. PTHrP regulates calcium homeostasis during lactation in a classic endocrine feedback loop. PTHrP is secreted from the mammary epithelial cells (MEC) into the circulation and stimulates osteoclastic and osteocytic bone resorption from the skeleton. The free calcium released into the bloodstream feeds back to the mammary gland where it activates the CaSR on the MEC to promote transepithelial transport of calcium into milk, and to inhibit further production and secretion of PTHrP into the circulation. MEC: Mammary epithelial cell, PTHrP: Parathyroid hormone-related peptide, CaSR: Calcium sensing receptor, RANKL: Receptor activator of NF-KB ligand, RANK: Receptor activator of NF-KB; Ca^2+^: Ionized calcium. Image made with BioRender.

The calcium sensing receptor (CaSR) is a G-coupled protein receptor (GPCR) that signals in response to extracellular calcium levels. Outside of lactation, the CaSR regulates PTH secretion from the parathyroid glands and calcium excretion by the renal tubules (Conigrave, 2016). While the CaSR is expressed at low levels in the mammary gland of virgin and pregnant animals, its expression increases many-fold in mammary epithelia during lactation, where its activation inhibits PTHrP secretion and stimulates transepithelial calcium transport into milk (VanHouten et al., 2004; Ardeshirpour et al., 2006). If calcium delivery to the mammary gland falls, then PTHrP production rises and calcium transport decreases. These effects, in turn, lower calcium usage by the gland and increase bone turnover from the maternal skeleton to liberate calcium into the bloodstream. Circulating calcium provides negative feedback to the mammary gland to suppress further PTHrP production but positive feedback to increase calcium transport. This endocrine and nutrient-sensing feedback loop between the lactating breast and bone coordinates the supply of calcium with the demands for calcium during milk production using the CaSR to regulate PTHrP levels and calcium transport by the mammary epithelia (Figure 2).

The model for the feedback loop between the mammary gland and bone during lactation was derived from a study of lactating CaSR^+/−^ mice as well as mice with mammary-specific deletion of the *Casr* gene (CaSR-cKO) at the start of lactation (Mamillapalli et al., 2013) (Figure 2). Loss of the CaSR on mammary epithelial cells led to increased PTHrP gene expression, increased milk PTHrP content and reduced milk calcium content. PTHrP bioactivity, measured by urinary cyclic AMP production was significantly elevated in CaSR-cKO animals and they developed hypercalcemia due to reduced calcium transport into milk. The elevated calcium levels were transient as circulating calcium levels were normalized as a result of a compensatory decrease in PTH secretion and an increase in urinary calcium excretion triggered, in part, by stimulation of the parathyroid and renal CaSR by elevated circulating calcium. Although rates of bone loss measured by DXA and bone mass by microCT both were equivalent in CaSR-cKO animals as compared to lactating counterparts, histomorphometry revealed reduced trabecular bone volume, increased trabecular spacing and a decrease in trabecular number suggesting slightly excessive bone loss. There are no corresponding genetic experiments in human subjects but the data from mice confirm that, during lactation, upregulation of CaSR expression allows the mammary gland to become a calcium-sensing organ and the CaSR regulates PTHrP production and calcium transport in response to changes in calcium delivery to the gland.

### Prolactin: Mammary gland to brain to bone

Prolactin is a peptide hormone responsible for promoting breast development, secretory differentiation of breast epithelial cells, and milk production. Classically, prolactin is synthesized and secreted by the anterior pituitary under dopamine-mediated hypothalamic regulation. However, other tissues including the central nervous system, the immune system, the uterus, and the mammary gland are capable of producing prolactin (Al-Chalabi et al., 2022). With the fall in progesterone at parturition, prolactin receptors on the mammary alveolar cells are increased, enabling lactogenesis. Suckling or nipple stimulation is the most potent stimulator of prolactin production and secretion. Sensory nerves in the nipple carry the mechanical stimulus signal *via* the spinal cord to the arcuate nucleus where dopamine release is inhibited resulting in removal of its inhibitory effect on prolactin secretion. Simultaneously, afferent signals from nipple stimulation relay to the supraoptic and paraventricular nuclei to increase the production of oxytocin, responsible for milk let down. Increased oxytocin levels suppress dopamine and allow for further production of prolactin (Al-Chalabi et al., 2022). To control milk production, prolactin levels spike with periods of nipple stimulation. While suckling is maintained, prolactin levels continue to remain elevated. When suckling stops, prolactin levels decline to a basal level and milk production stops (Grosvenor and Whitworth, 1974; Freeman et al., 2000; Al-Chalabi et al., 2022). If a mother does not continue to nurse her baby, prolactin levels will fall to non-pregnant levels in 1 to 2 weeks.

Aside from its role in milk production and mammary gland development, prolactin integrates lactation physiology with bone metabolism. In mammary tissue, prolactin stimulates PTHrP production which, as noted above, increases bone turnover for delivery of calcium towards milk production. Prolactin has been shown to increase the genes essential for serotonin synthesis resulting in increased serotonin levels in the mammary epithelium and in milk (Matsuda et al., 2004). Serotonin, in turn, has been shown to affect mammary epithelial cell differentiation, alveolar filling and PTHrP production (Matsuda et al., 2004; Hernandez et al., 2012; Zang et al., 2018). Several studies in mice, rats, goats, sheep and cows have demonstrated that administration of serotonin precursors in lactating animals increases circulating levels of calcium and PTHrP as well as milk calcium concentrations and bone resorption (Hernandez et al., 2012; Laporta et al., 2013; Laporta et al., 2014; Jin et al., 2019; Zhang et al., 2022). In addition, blocking serotonin synthesis by knocking out the tryptophan hydroxylase 1 gene in mice lowered PTHrP gene expression in the lactating mammary gland (Laporta et al., 2014). Finally, it appears that serotonin induces PTHrP expression, in part through a hedgehog-dependent pathway (Laporta et al., 2014). These data suggest that prolactin can regulate bone turnover indirectly by inducing mammary serotonin production, which, in turn stimulates PTHrP production.

High serum prolactin levels also contribute to the low estrogen state of lactation. In both animals and in women, prolactin can inhibit pulsatile GnRH release by the hypothalamus. In mice and rats, this has been shown to occur through altering tuberoinfundibular dopamine turnover, and also inhibiting kisspeptin 1 secretion (Smith and Grove, 2002; Smith et al., 2010; Brown et al., 2014). This reinforces the direct actions of the suckling reflex (discussed in prior section) reinforcing hypogonadotropic hypogonadism and low estrogen levels. Prolactin has also been shown to directly inhibit estradiol production by ovarian granulosa cells in response to FSH, again reinforcing the reduction in circulating estrogen concentrations (McNeilly et al., 1982; Nakamura et al., 2010). Thus, prolactin also can increase bone resorption and calcium mobilization by contributing to the hypogonadism induced by suckling.

In addition to prolactin’s indirect effect on bone turnover *via* lowering estrogen and raising PTHrP levels, it may also have a direct effect on osteoblasts which express prolactin receptors. Mice with prolactin receptor deficiency have decreased bone formation (Clément-Lacroix et al., 1999; Coss et al., 2000; Seriwatanachai et al., 2008) and rats treated with bromocriptine to lower prolactin levels had a blunted bone mineral density gain related to pregnancy (Suntornsaratoon et al., 2009; Suntornsaratoon et al., 2010). Hyperprolactinemia resulting from anterior pituitary implantation increases the RANKL/OPG ratio, favoring osteoclastic resorption *in vivo* and decreases the mRNA expression of bone mineralization markers such as osteocalcin and alkaline phosphatase in osteoblast cell culture (Seriwatanachai et al., 2008). These direct responses to prolactin may lead to the suppression of bone formation. Furthermore, prolactin controls intestinal calcium absorption by increasing active transcellular and passive calcium transport in the small intestine during pregnancy and lactation (Pahuja and DeLuca, 1981; Charoenphandhu and Krishnamra, 2007; Charoenphandhu et al., 2009).

### Oxytocin

Suckling stimulates the secretion of oxytocin from the posterior pituitary. This neuropeptide hormone is well known to regulate milk ejection and to contribute to social bonding between mothers and infants. Moreover, oxytocin is synthesized in the bone microenvironment and has direct effects on bone cells. Osteoblasts, osteoclasts, and both osteoblast and osteoclast progenitors express oxytocin receptors (Colucci et al., 2002). Oxytocin can stimulate the expansion of osteoclast progenitors while also inhibiting mature osteoclast function. However, any effects on bone resorption appear to be mitigated by the more dominant effects to stimulate osteoblasts and bone formation (Liu et al., 2009; Tamma et al., 2009). Disruption of either the oxytocin gene or the oxytocin receptor gene results in osteoporosis and administration of oxytocin to ovariectomized mice prevents bone loss caused by estrogen withdrawal (Liu et al., 2009; Tamma et al., 2009; Beranger et al., 2015). Thus, on balance, it has been suggested that oxytocin has primarily an anabolic function on bone. However, Sun and colleagues found that the specific targeted deletion of the oxytocin receptor in osteoblasts resulted in attenuation of lactation-induced bone loss suggesting that oxytocin actions on osteoblasts somehow increased bone loss, which is the opposite of its effects in the ovariectomy model and its known effects to increase osteoblast RANKL expression (Sun et al., 2016). Unfortunately, the authors did not characterize bone turnover markers or histomorphometric measurements of bone cell populations in these experiments, so the mechanisms underlying these observations remain unclear. It is possible that oxytocin may be another reinforcing factor that contributes to increased bone turnover and bone loss during lactation.

### Calcitonin

Calcitonin is a peptide hormone that is primarily produced by the parafollicular cells of the thyroid gland and acts to reduce circulating calcium levels by directly inhibiting the function of osteoclasts. The presence of calcitonin and its receptor in mammary tissue (Tverberg et al., 2000), bone and the pituitary (Sheward et al., 1994; Ren et al., 2001) suggests that calcitonin may also participate in the regulation of calcium homeostasis during lactation. In mice, it has been shown that calcitonin protects the maternal skeleton from excessive bone loss during lactation. Calcitonin null mice lost 53% of their bone mineral content in the spine compared to wildtype control mice that lost the expected 26% BMC during lactation (Woodrow et al., 2006). This excess bone loss could be rescued by administration of salmon calcitonin, the pharmacological form used to treat bone diseases. Global knockout of the calcitonin receptor in mice revealed that calcitonin limits osteocytic osteolysis during lactation by regulating acidification of the osteocyte lacunae. Lower pH in the osteocyte lacunae and higher levels of gene expression of the ATPase H+ transporting V0 subunit D2 (Atp6v0d2) were reported in mice lacking the calcitonin receptor gene. Finally, calcitonin null mice fully recover bone mass after weaning to levels consistent with wildtype animals, demonstrating that it is not required for the anabolic effects of weaning (Woodrow et al., 2006).

The effect of calcitonin on the pituitary and mammary gland during lactation are less clear cut. Exogenous calcitonin administration to lactating rats (Tohei et al., 2000) and targeted overexpression of calcitonin in pituitary lactotrophs in mice (Yuan et al., 2005) inhibited prolactin production. Although it was expected that calcitonin null mice may have enhanced synthesis of prolactin and even more depressed levels of estrogen contributing to the exaggerated bone loss during lactation, there was no significant mean difference in circulating prolactin levels between calcitonin null animals and wildtype, and estrogen levels were undetectable in both groups (Woodrow et al., 2006). In the mammary gland, where calcitonin and its receptor are both expressed (Tverberg et al., 2000), calcitonin may regulate PTHrP production. Calcitonin null mice have higher levels of PTHrP during lactation compared to wildtype, but there are no local changes in the gene expression of the CaSR, which typically regulates PTHrP production as described above, or in the gene expression of calcium regulators or calcium handling proteins involved in milk production (Woodrow et al., 2006). This suggests that the effect of calcitonin on PTHrP production may be through regulation of CaSR activity or independent of the CaSR.

There are no controlled studies in human examining the effect of calcitonin on lactation-associated bone loss. Future studies on women with low calcitonin levels as a result of having had a total thyroidectomy prior to pregnancy may shed light on the role of calcitonin in skeletal remodeling with breastfeeding.

### Pregnancy and lactation-associated osteoporosis

Remarkably, the maternal skeleton can undergo multiple rounds of significant bone loss and replenishment with successive periods of pregnancy and lactation without clinical consequence. Pregnancy and lactation associated osteoporosis (PLO) is a rare condition where women develop fragility fractures associated with significantly low BMD during pregnancy or the postpartum period. The incidence rate has been estimated to be 4–8 per 1 million pregnancies. Much of what is known about PLO derives from observational studies and clinical case reports or case series, in which little is known about the mother’s pre-pregnancy or pre-fracture bone quality.

Vertebral fractures occur most commonly in PLO and are considered the hallmark of this disorder. Furthermore, PLO is often associated with multiple vertebral fractures, with an average of three, but up to eight reported in a single patient (Bonacker et al., 2014; Hadji et al., 2017; Hardcastle et al., 2019). The most vulnerable vertebral bodies appear to be T11-L4. The high prevalence of multiple vertebral fractures in PLO may reflect reporting bias as asymptomatic single vertebral fractures may be underreported or underdiagnosed. Nevertheless, women with PLO usually present with back pain in late pregnancy or during early lactation. Other low-trauma fractures have also been reported in PLO including hip, pelvic, sacral, pubic, humeral, tibial and foot fractures (Breuil et al., 1997; Aynaci et al., 2008; Laroche et al., 2017; Kyvernitakis et al., 2018; Cohen et al., 2019; Jun Jie et al., 2020; Tuna et al., 2020).

Limited studies have looked at bone density and structure in PLO. Most data have been collected near the time of fracture or remotely after the fracture without clear relation to the timing of breastfeeding. Data from published case series have shown that women with PLO have significantly reduced BMD by DXA, with lower values in the spine than the total hip (Hardcastle et al., 2019; O'Sullivan et al., 2006; Hong et al., 2018). Spine z-scores are often −3 or lower, while total hip z-scores are generally in the −2 range. Bone structure, as assessed by HR-pQCT, shows reduced cortical density and thickness, as well as reduced trabecular number, density and thickness in women with PLO as compared to young, healthy women (Sánchez et al., 2016). A small number of studies included bone histology from transiliac crest biopsies of women with PLO. In a study by Smith et al. of eleven women with PLO, bone biopsies were obtained 2 weeks to 5 years post-delivery. Four biopsies showed normal bones. Six biopsies showed osteoporosis without evidence of increased bone activity or osteoid. One biopsy revealed underlying osteogenesis imperfecta. One biopsy had evidence of increased osteoblastic and osteoclastic activity; however this sample was obtained 5 years postpartum. Despite the heterogeneity, the authors suggested that PLO is a condition of reduced osteoblastic activity rather than increased bone resorption (Smith et al., 1995). Cohen et al. performed bone biopsies on seven women with PLO and compared them to women with premenopausal osteoporosis unrelated to pregnancy or lactation, and to healthy women. All bone biopsies from women with PLO were taken at least 12 months after delivery and six or more months after weaning from breastfeeding. They found that women with PLO had significantly lower rates of bone apposition and formation compared to the other two groups. However, osteoblast numbers were similar among all the groups suggesting that PLO may be a condition with a deficit in osteoblast function. In contrast, a bone biopsy taken 10 weeks postpartum from a woman with PLO showed features of increased bone turnover including increased numbers of osteoblasts, osteoclasts, and eroded surfaces (Cohen et al., 2019). These differences in timing of bone biopsies relative to pregnancy and lactation, make it difficult to tease out the underlying causes that are contributing to increased skeletal fragility in PLO. A recent genetics study of 42 women with PLO from a referral center in Germany found that 50% of patients had genetic variants of genes known to play a role in bone formation, supporting the idea that PLO may represent a condition of “sluggish” osteoblasts that may not be able to mount enough of a response to match the normal increase in bone formation seen in lactation, resulting in a larger or more rapid net loss of bone mass in response to all of the changes that drive bone resorption during this period (Kyvernitakis et al., 2018).

For women presenting with fracture in pregnancy or lactation, it is important to have a high clinical index of suspicion for PLO and to identify any underlying causes of bone fragility and osteoporosis that are being unmasked by the physiology of pregnancy and lactation. In addition to a full history and physical exam, it is important to take a full dietary history including calcium intake. A history of fragility fractures prior to pregnancy or multiple fractures during childhood, joint hypermobility or blue sclerae should prompt further evaluation for osteogenesis imperfecta. A personal or family history of visual impairment warrants further investigation for mutations in the low-density lipoprotein-related receptor 5 (LRP5), which causes osteoporosis-pseudoglioma syndrome. Assuming that BMD is low, secondary causes of osteoporosis should be considered including blood screen with full blood count, calcium profile, renal function, vitamin D and thyroid stimulating hormone. If clinical features are suggestive, screening for Cushing’s syndrome or multiple myeloma may be appropriate. Bone turnover markers are often collected but have limited utility in the acute setting of fracture.

The approach to treating women with PLO remains controversial and will not be discussed in detail here. Antiresorptive and anabolic agents used for the treatment of postmenopausal osteoporosis have been used as therapy for women with PLO even though they are all considered contraindicated in pregnancy and lactation. The conservative approach of ensuring adequate calcium and vitamin D intake appears to be of low risk. It is reasonable to counsel women with PLO and very low bone density to stop breastfeeding, given that BMD is expected to decrease by 1%–3% per month of lactation. Some evidence exists that breastfeeding in the face of PLO may increase the risk of fractures in subsequent pregnancies (Laroche et al., 2017; Kyvernitakis et al., 2018; Hardcastle et al., 2019). Bisphosphonates have been well tolerated in women with PLO but potential safety concerns in women of childbearing age must be acknowledged since it is known that bisphosphonates have long skeletal retention times and readily cross the placenta. Denosumab has been used for treatment of PLO and is attractive for its quick offset time compared to bisphosphonates (Ijuin et al., 2017; Cerit and Cerit, 2020; Tuna et al., 2020). However, the rebound increased bone turnover known to occur on discontinuation of denosumab is a potential issue, particularly in women who have already sustained vertebral fractures. More frequently, anabolic agents are used to treat PLO and have demonstrated clinical improvement in pain and increases in BMD (Stumpf et al., 2007; Hellmeyer et al., 2010; Choe et al., 2012; Lampropoulou-Adamidou et al., 2012; Lee et al., 2013; Bonacker et al., 2014; Pola et al., 2016; Ijuin et al., 2017; Laroche et al., 2017; Hong et al., 2018; Cerit and Cerit, 2020; Tuna et al., 2020). Two recently published studies have demonstrated that use of teriparatide results in improved bone density as compared to calcium and vitamin D alone (Lee et al., 2021; Hadji et al., 2022). It is also notable that the study from Lee and colleagues demonstrated that teriparatide could be used in this setting to increase bone density and that subsequent use of an antiresorptive medication may not be necessary as patients who did not receive an anti-resorptive agent did not lose bone for up to 2 years after discontinuing anabolic therapy with PTH (Lee et al., 2021). These studies suggest that teriparatide should be the preferred therapy for patients with PLO who require treatment. One caveat is that these studies did not examine fractures, they just monitored changes in bone density measurements. To date, there are no reported cases of romozosumab use for PLO. Clearly more study is required to better understand the underlying pathophysiology and best treatment for this rare but devastating condition.

### Lactation as an evolutionary template for bone loss in postmenopausal osteoporosis

Postmenopausal bone loss has been theorized to be an inappropriate reactivation of the mechanisms designed for physiological bone loss during lactation. These two reproductive phases share multiple similarities. First, there is uncoupled bone turnover leading to net bone loss, which is more prominent at trabecular-rich sites. Second, estrogen deficiency is one of the main drivers for unbalanced bone resorption (Kovacs and Kronenberg, 1997; Wysolmerski, 2002). The ability to store and mobilize calcium during lactation may underlie the skeleton’s estrogen responsiveness. If this is true, then menopausal bone loss can be regarded as a post-reproductive consequence of the increase in bone resorption that normally occurs during lactation in response to estrogen deficiency. Understanding the mechanisms that mediate full skeletal recovery post-weaning may thus lead to clinical interventions for the reversal of osteoporosis.

## Conclusion

Coordination of the brain-breast-bone axis during lactation ensures an adequate supply of calcium for milk production that, in turn, supports the growth and development of mammalian offspring. This circuitry contains feedback loops and redundancies that serve to balance the increased metabolic demands for calcium to supply milk production with the maintenance of skeletal integrity to allow for multiple reproductive cycles. Much of our understanding of this brain-breast-bone circuity is based on rodent models. Clinical studies with bone biopsies taken during lactation are needed to understand the cellular and microstructural changes that occur in humans as well as to determine whether osteocytic osteolysis also occurs in nursing humans. With this knowledge, we may be able to enhance our approach to the treatment of pregnancy and lactation associated osteoporosis and/or to develop new therapies and targets for postmenopausal osteoporosis.
